# Determination of the Porosity of PLGA Microparticles by Tracking Their Sedimentation Velocity Using a Flow Imaging Microscope (FlowCAM)

**DOI:** 10.1007/s11095-017-2120-8

**Published:** 2017-02-17

**Authors:** A. S. Sediq, S. K. D. Waasdorp, M. R. Nejadnik, M. M. C. van Beers, J. Meulenaar, R. Verrijk, W. Jiskoot

**Affiliations:** 1 Division of Drug Delivery Technology, Cluster BioTherapeutics, Leiden Academic Centre for Drug Research (LACDR), Leiden University, Leiden, The Netherlands; 2 Dr. Reddy’s Laboratories Ltd., IPDO, Leiden, The Netherlands

**Keywords:** flow imaging microscope, FlowCAM, particle analysis, PLGA microparticles, porosity

## Abstract

**Purpose:**

To investigate whether particle sedimentation velocity tracking using a flow imaging microscope (FlowCAM) can be used to determine microparticle porosity.

**Methods:**

Two different methods were explored. In the first method the sedimentation rate of microparticles was tracked in suspending media with different densities. The porosity was calculated from the average apparent density of the particles derived by inter- or extrapolation to the density of a suspending medium in which the sedimentation velocity was zero. In the second method, the microparticle size and sedimentation velocity in one suspending fluid were used to calculate the density and porosity of individual particles by using the Stokes’ law of sedimentation.

**Results:**

Polystyrene beads of different sizes were used for the development, optimization and validation of the methods. For both methods we found porosity values that were in excellent agreement with the expected values. Both methods were applied to determine the porosity of three PLGA microparticle batches with different porosities (between about 4 and 52%). With both methods we obtained microparticle porosity values similar to those obtained by mercury intrusion porosimetry.

**Conclusions:**

We developed two methods to determine average microparticle density and porosity by sedimentation velocity tracking, using only a few milligrams of powder.

**Electronic supplementary material:**

The online version of this article (doi:10.1007/s11095-017-2120-8) contains supplementary material, which is available to authorized users.

## Introduction

Formulating active pharmaceutical ingredients (API) in controlled release systems is a potent strategy to maintain drug levels for prolonged periods within the therapeutic window, which may increase the efficiency of therapy, reduce the costs and improve patient compliance and comfort (1). Owing to their long clinical experience and favorable performance in terms of biodegradability and biocompatibility, PLGA microparticles fulfill the needs for controlled release in the area of parenteral pharmaceutical formulations, with a number of FDA approved drug products on the market today (2).

The porosity or void fraction of poly lactic-co-glycolic acid (PLGA) microparticles is a critical parameter known to affect the release kinetics of encapsulated drugs (3–5). Current approaches to determine the porosity of particulate drug delivery systems are based on established methods used in agricultural, petrochemical and constructional engineering (6). Among the available methods, mercury intrusion porosimetry (MIP) and gas (nitrogen) adsorption based methods are the most common and informative ones, because both can measure the pore size and its distribution. In addition, mercury porosimetry has the advantage of having certified reference material and standard measurement protocols (7). However, each of these methods has a number of major drawbacks. For instance, both methods require large amounts of sample (200–300 mg) for a single measurement. In addition, with MIP the difficulties are seen in distinguishing intra- and interparticulate pores (8). Besides, presence of enclosed pores may need additional MIP measurements with grinded material (9) and ink-bottle shaped and interconnected pores can lead to underestimation of the pore size (8). Toxic metal waste is yet another reason that would make the application of MIP less favorable. In contrast to MIP, with gas adsorption methods both open and enclosed pores are measured; however, the process of pressure equilibration may be very slow, resulting in long-lasting measurements for a single sample. Last but not least, although fully automated equipment is commercially available for both methods, such equipment is expensive and not available in many pharmaceutical laboratories.

Taking into account the drawbacks of the methods described above, there is need for a more straightforward method requiring small amounts of sample for deriving the overall porosity of (pharmaceutical) particulate systems. Considering the developments in flow imaging instruments with respect to image quality, sizing precision and accuracy (10) and their increasingly widespread use in pharmaceutical laboratories, we have evaluated sedimentation velocity tracking using a flow imaging microscope (FlowCAM) for measuring the density and porosity of PLGA microparticles. To our knowledge, in spite of the simplicity of the concept, a sedimentation based approach has not been used before to measure particle porosity. The velocity of a settling particle depends among other parameters on the size of the particle and the density difference between the liquid and the particle (11). The density of each component of a microparticle (e.g., PLGA matrix, drug, and liquid or air filling the pores) contributes proportionally to the total microparticle density, and can therefore be used to calculate the porosity of a microparticle.

Here we present two methods using a FlowCAM to determine the porosity of PLGA microparticles. The results show that both methods generate porosity values close to those obtained with MIP, but require much smaller amounts of sample.

## Materials and Methods

### Materials

Cesium chloride (CsCl), polysorbate 80 and ethanol were obtained from Sigma (Sigma-Aldrich, Steinheim, Germany). Phosphate buffered saline (PBS; 8.2 g/L NaCl, 3.1 g/L Na_2_HPO_4_.12H_2_O, 0.3 g/L NaH_2_PO_4_.2H_2_O, pH 7.4) was purchased from Braun (B. Braun Melsungen AG, Germany) and filtered with a 0.22-μm polyethersulfone-based syringe-driven filter unit (Millex GP, Millipore, Carrigtwohill, Ireland). Ultrapure water (18.2 MΩ.cm) was dispensed by using a Purelab Ultra water purification system (ELGA LabWater, Marlow, UK). Non-porous polystyrene sizing standards of different sizes (29.8 ± 0.4, 50.2 ± 0.5 and 69.1 ± 0.8 μm) were purchased from Duke Scientific (through Thermo Scientific, Fremont, CA, USA). Three batches of dried PLGA microparticles were kindly provided by Dr. Reddy’s Laboratories Ltd. (IPDO, Leiden, the Netherlands). One of these batches (batch 1) contained API. The other two batches (2 and 3) were loaded with different amounts of an API. The microparticle batches had different porosities as measured with MIP, namely 4.0, 21.6, and 51.9% for batch 1, 2 and 3, respectively. The residual water content and residual organic solvent content of each PLGA microparticle batch were found to be lower than 0.5% (*w*/*w*) and were not taken into account.

### Surface Morphometry Using Scanning Electron Microscopy

Scanning electron microscopy (SEM, Nova NanoSEM, FEI, Eindhoven, the Netherlands) was used for high resolution imaging of the surface of PLGA microparticles. Microparticles were coated with a thin layer of gold in order to increase the surface conductivity. The instrument was operated at 15 kV and images were taken at magnifications between 50 and 400×.

### Sample Preparation for Sedimentation Velocity Tracking

Solutions of PBS containing 0.01% (*w*/*v*) polysorbate 80 (PBS-T) were prepared with varying fluid densities by adding different concentrations of CsCl. Polysorbate 80 was included to facilitate wetting of the microparticles. The concentration of CsCl ranged from 0–75% (*w*/*w*), resulting in fluid densities ranging from about 1000–1655 kg/m^3^. The density and viscosity of the used suspending fluids were observed to be dependent on the concentration of CsCl, and were taken into account in further calculations. All the measurements were performed at room temperature. A few drops of the concentrated polystyrene sizing standards were added to 10 mL of the PBS-T/CsCl solutions. For each polystyrene standard suspension, the sedimentation of 50–100 particles was tracked using FlowCAM. In order to study PLGA microparticle sedimentation, an appropriate amount of microparticles was suspended in PBS-T/CsCl to achieve a microparticle concentration of about 0.25 mg/mL (corresponding to approximately 7000–15,000 particle counts/mL). These relatively low particle concentrations were chosen in order to avoid physical agglomeration and optical coincidence of settling particles. After addition of the suspending medium to the microparticles, the suspension was sonicated for 20 min and left at ambient conditions for at least 3 h prior to analysis.

### FlowCAM set-up for Sedimentation Velocity Tracking

A FlowCAM VS1 system (Fluid Imaging Technologies, Yarmouth, ME, USA) equipped with a 300-μm Field of View (FOV300; 300 μm depth and 1500 μm width) cell and 4× magnification lens was used in this study. VisualSpreadsheet software version 3 was used to control the system and to process the data. Prior to each measurement, the flow cell was rinsed with 2 mL particle-free suspending medium corresponding to the sample being measured. The background was calibrated by manually priming 0.5 mL of the same particle-free suspending medium. Hereafter, 1.5 mL of the sample was loaded and FlowCAM measurement was started with a flow rate of 0.20 mL/min and a camera rate of 10 frames/s. As soon as the sample had completely filled the flow cell and tubing (based on the volume estimated from the flow cell and tubing dimensions), the tubing was disconnected from the pump and both tubing ends were clamped to create a closed system in which there is no liquid flow. The analysis was stopped manually as soon as a sufficient number of particles was tracked (50 – 100 particles). The sample volume was set to 10 mL in the software settings to avoid premature, automatic termination of the analysis.

### Sedimentation Velocity from FlowCAM Data

In order to optimize the measurement and to minimize the risk of tracking impurities or particles with anomalous settling behavior, the following particle inclusion criteria were used:

1. Edge gradient (average intensity of the pixels making up the outside border of a particle) values between 100–200 a.u. and aspect ratio values above 0.9. This criterion selects only particles that are in focus.
2. A distance of at least twice the diameter between the left/right edge of the particle and vertical sides of the flow cell (determined with the help of X-coordinate and the known width of the field of view). This criterion discards particles that undergo retardation in velocity due to the left and right edges of the flow cell.
3. A straight vertical movement path of the particle found when the X- and Y-coordinates at each image is plotted (i.e., X-coordinate does not change more than 5 pixels during the entire track).
4. A constant particle displacement as a function of time.

In this way, only accurately sized particles without any unordinary settling motion were used for sedimentation velocity tracking.

The particles with properties that met the aforementioned criteria were extracted from the entire set of raw data. The Y-coordinate values (expressed in pixels) were converted to metric distances, with the use of image scale (named calibration factor in VisualSpreadsheet). After plotting the time (in seconds) against metric displacement, the velocity was found as slope of the linear regression with the help of Excel 2010 software.

In addition, the average values for properties such as area based diameter (ABD, the diameter based on a circle with an area that is equal to the projected particle area) and aspect ratio were extracted for each tracked particle.

### Method I: PLGA Microparticle Porosity from Sedimentation Velocity in Fluids with Varying Densities (Density-Matching Method)

The first approach that was used to derive the density and, subsequently, the porosity of analyzed particles consisted of tracking the individual particle sedimentation velocity in fluids with different densities. For each bead or microparticle suspension, the sedimentation velocity of individual particles was derived as described above. In order to normalize the derived velocities (v, in m/s) for particle size, they were divided by the corresponding average square diameter (calculated from ABD values; d^2^, in m^2^). The resulting particle size-normalized sedimentation velocity (v/d^2^, in m^−1^s^−1^) values were based on the relation between velocity and diameter found in Stokes’ law of sedimentation, as shown in Eq. 1:

1 $$ v=\frac{\left({\rho}_p-{\rho}_f\right)}{18\mu} g{d}^2 $$

where ρ_p_ and ρ_f_ are the particle and fluid densities (in kg/m^3^), respectively, μ is the dynamic viscosity of the fluid (in kg/m.s) and g is the gravitational acceleration (m/s^2^). Note that (particle size-normalized) sedimentation velocity values will be positive for settling particles and negative for floating particles.

Subsequently, the average v/d^2^ values for each bead size or PLGA microparticle batch in different suspending media were plotted against the density of the corresponding suspending medium. Assuming that the particle would stagnate when its density is equal to that of the liquid, the intercept of a linear regression with the X-axis (density axis) was taken as the average particle density of the concerning polystyrene bead or PLGA microparticle batch. In case of a porous particle containing an API, the particle density will be the sum of the fractional densities of (i) the matrix (f_PLGA_ × ρ_PLGA_), (ii) the API (f_API_ × ρ_API_) and (iii) the pores (f_pore_ × ρ_pore_). Assuming that the pores are filled with air (i.e., ρ_pore_ = 0; further explained in the “Discussion” section), Eq. 2 was used to calculate the density derived particle porosity (φ in%) (6):

2 $$ \varphi =\frac{\rho_{solid}-{\rho}_p}{\rho_{solid}}100\% $$

where ${\rho}_{solid}={f}_{API}\times {\rho}_{API}+{f}_{PLGA}\times {\rho}_{PLGA}$ in which ρ_solid_ is the density of the solids, i.e., API and the PLGA matrix, and f_API_ and f_PLGA_ are the weight fractions of the API and the PLGA, respectively, in the solids content (derived from the drug loading in percent).

### Method II: PLGA Microparticle Porosity from Sedimentation Velocity Using Stokes’ law (Stokes Derived Method)

For the second method only the particles tracked in a suspending fluid having a density close, but not equal, to the expected particle density were used. As compared to high sedimentation velocities, low sedimentation velocities result in more accurate density determinations, because of the large number of 2D-coordinate data points gained from a large number of images taken during the particle tracking time lapse. Subsequently, the density and resulting porosity of individual particles were calculated, by means of Stokes’ law.

Bach *et al*. derived equations for calculating the density of a settling particle in a fluid (12). Here, we used the same approach, but with porosity of the particle as the final outcome. The Stokes’ law of sedimentation was used as a starting point for the calculations. This formula gives a mathematical description for the drag force exerted on spherical objects when the Reynolds number is very small (Re < < 0.1) (13). The Reynolds numbers associated with the relative flow of particles tracked in our study were calculated to be < < 0.01, using Eq. 3:

3 $$ R e=\frac{{vd\rho}_f}{\mu} $$

Incorporating equivalent of ρ_p_ from Eq. 2 in Eq. 1, and rewriting it to porosity (%) leaves us with the following:

4 $$ \varphi =\frac{\rho_{solid}-\left({\rho}_f=\frac{18\mu v}{g{ d}^2}\right)}{\rho_{solid}}100\% $$

In our calculations, in case of particle diameters equal to or larger than 50 μm we also applied a correction for the sedimentation velocity with respect to the retarding effect by the presence of the FlowCAM flow cell wall:

5 $$ {v}_{wc}=\frac{v_{measured}}{1- k\left(\frac{d}{D}\right)} $$

Here, the experimentally measured velocity (v_measured_) is corrected for coefficient of drag (k), particle diameter (d) and the shortest distance of the particle edge and the wall (D), to eventually gain the velocity corrected for the wall effect (v_wc_). The coefficient of drag depends on the shape of the space where settling takes place, and had a value of 1.004 (12).

We also took into account the effect of particle shape on the sedimentation velocity. For this purpose, based on the FlowCAM and SEM images, we have used the corrections specifically for prolate ellipsoid shapes (14):

6 $$ v={v}_{wc}\frac{\frac{8}{3}\left({\beta}^2-1\right)}{\frac{\beta \left(3{\beta}^2-2\right)}{{}_{\left({\beta}^2-1\right)}\frac{1}{2}}{ \tan}^{-1}\left({\left({\beta}^2-1\right)}^{\frac{1}{2}}\right)-\beta} $$

where β is the reciprocal aspect ratio. As seen in Eq. 6, the final velocity (v) is achieved by correcting the wall-effect-corrected velocity (v_wc_) using the average aspect ratio of each particle from the analyzed FlowCAM data.

## Results

### Sedimentation Velocity Data Processing and Analysis set-up Using Polystyrene Beads

FlowCAM was used to determine the sedimentation velocity of microparticles in aqueous liquids. The velocity value was then used to derive the density of the microparticles. From the density and known composition of the microparticles the porosity was calculated. Derivation of the sedimentation criteria to include a particle in the density calculations, corrections for influential parameters on the sedimentation (e.g., wall and shape effect) and the validation of the method were studied and performed by using polystyrene beads of three different sizes. In the supplementary document the development of the first selection criterion (Supplementary Figure S1) and the wall correction (Supplementary Figure S2) are explained in detail.

**Fig. 1:**
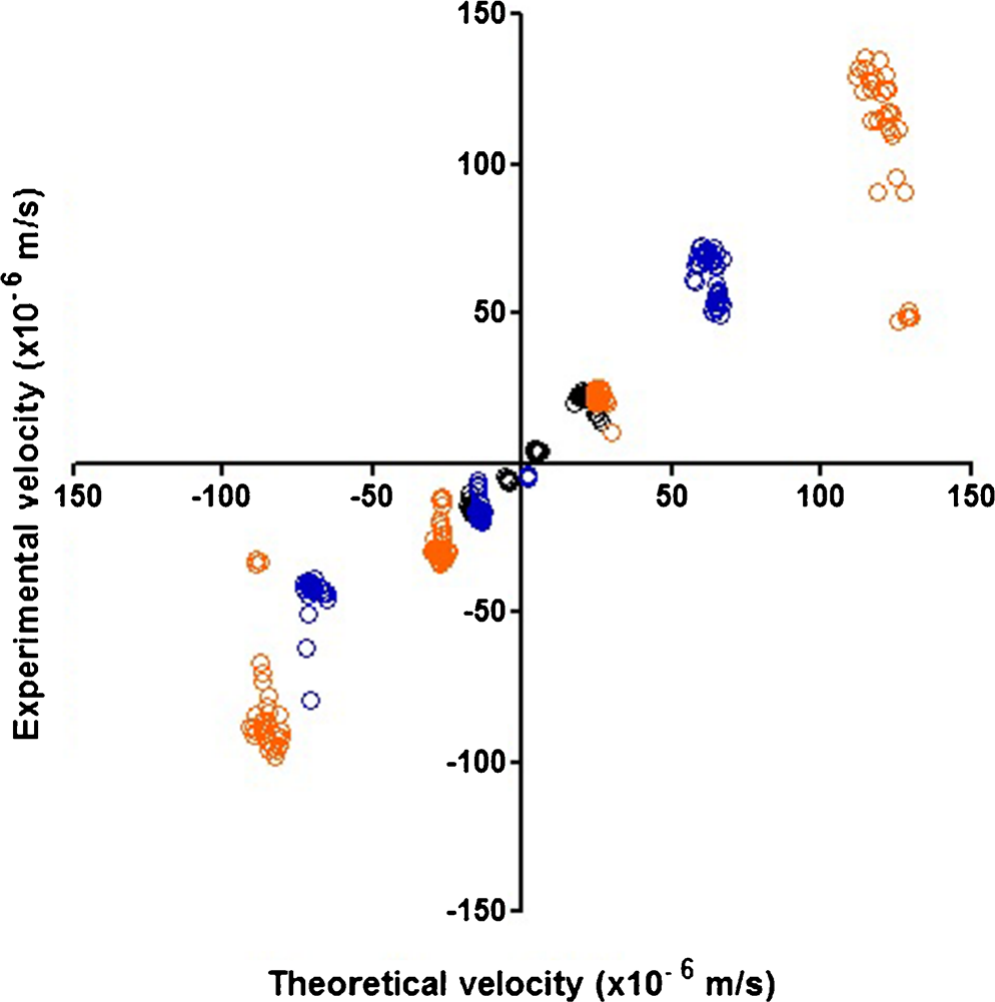
Theoretical sedimentation velocities plotted against experimental sedimentation velocities of polystyrene size standards suspended in liquids with different densities: 30-μm beads (black circles), 50-μm beads (blue circles) and 70-μm beads (orange circles). For the 50-μm and 70-μm beads, a correction for the wall effect was applied. The theoretical sedimentation velocity was calculated by using the measured size of the concerning particle and the bead density (1050 kg/m^3^) as provided by the manufacturer.

Figure 1 shows that the experimental sedimentation velocities (corrected for the wall effect) of all the polystyrene beads are in excellent correspondence with the theoretical values. This indicates that determining the density through measurement of the sedimentation velocity using a FlowCAM is possible for particles widely ranging in size and density difference (with respect to the suspending liquid), when applying the inclusion criteria named in “Materials and Methods” section.

**Fig. 2:**
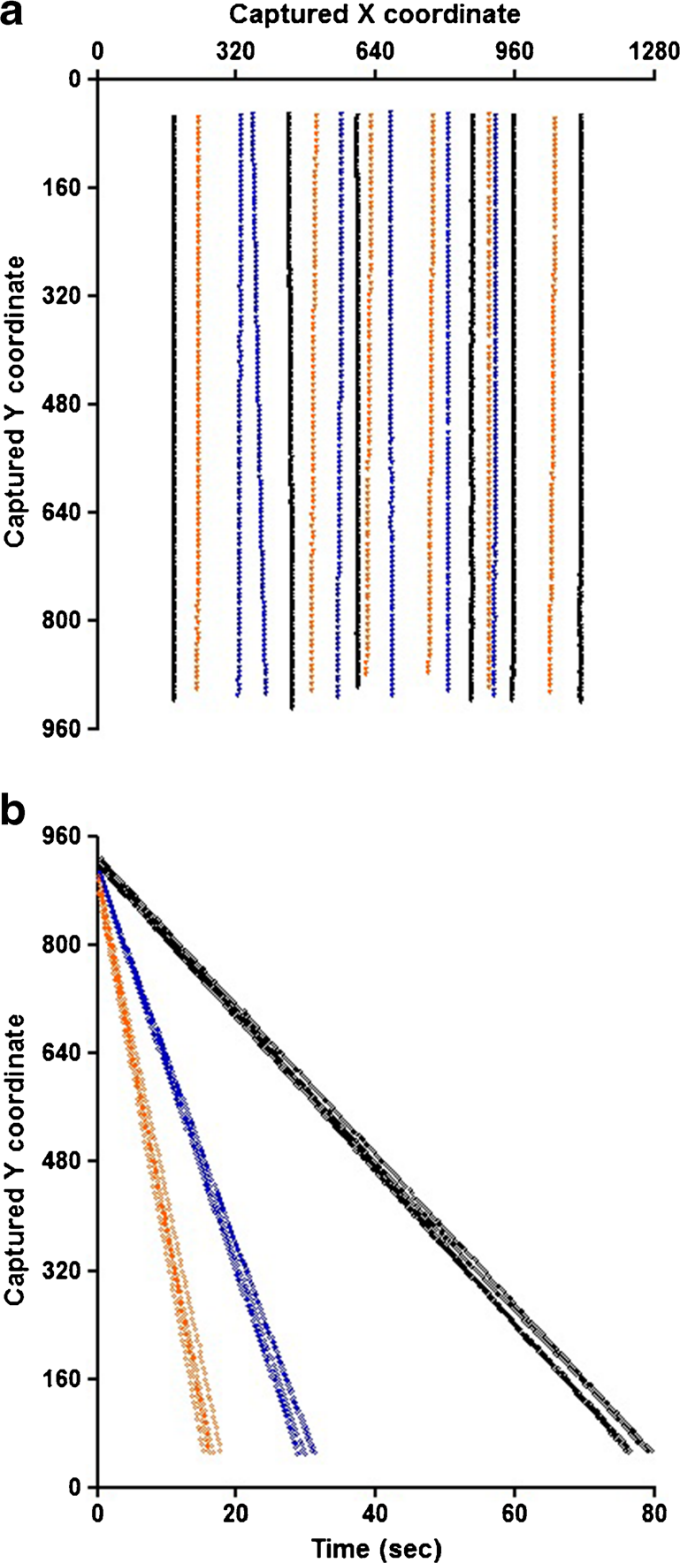
Polystyrene bead displacements in the 2-dimensional plain (graph A) and in time (graph B). The position of a settling particle in each image captured by FlowCAM is given by the X- and Y-coordinates in terms of pixel number from the lower left corner. This way the path of a settling particle can be derived, by making a scatter plot of the coordinates against the tracked time. This is illustrated in graph A for a number of beads of each polystyrene size standard (30-μm beads in black; 50-μm beads in blue; 70-μm beads in orange). In graph B the displacement in the Y-axis over time of the particles from graph A are shown. The slopes of these lines represent individual settling velocities (pix/s).

The complete data set of the particles fulfilling the inclusion criteria was extracted from the raw data and the physical displacement through the field of view was visualized by plotting the X- and Y-coordinates, as shown in Fig. 2a. At this point only the particles with a settling distance from the left or right flow cell border of at least two times the particle diameter and showing a vertical settling path were selected to be included in the analysis. In the last step of data processing, the vertical displacement was plotted against time, for individual particles, where the slope of the deduced line corresponds to the sedimentation velocity of the particle. Fig. 2b illustrates that the slopes, representing the sedimentation velocities, increase with increasing polystyrene bead size, as expected.

### Method Validation Using Polystyrene Beads

**Fig. 3:**
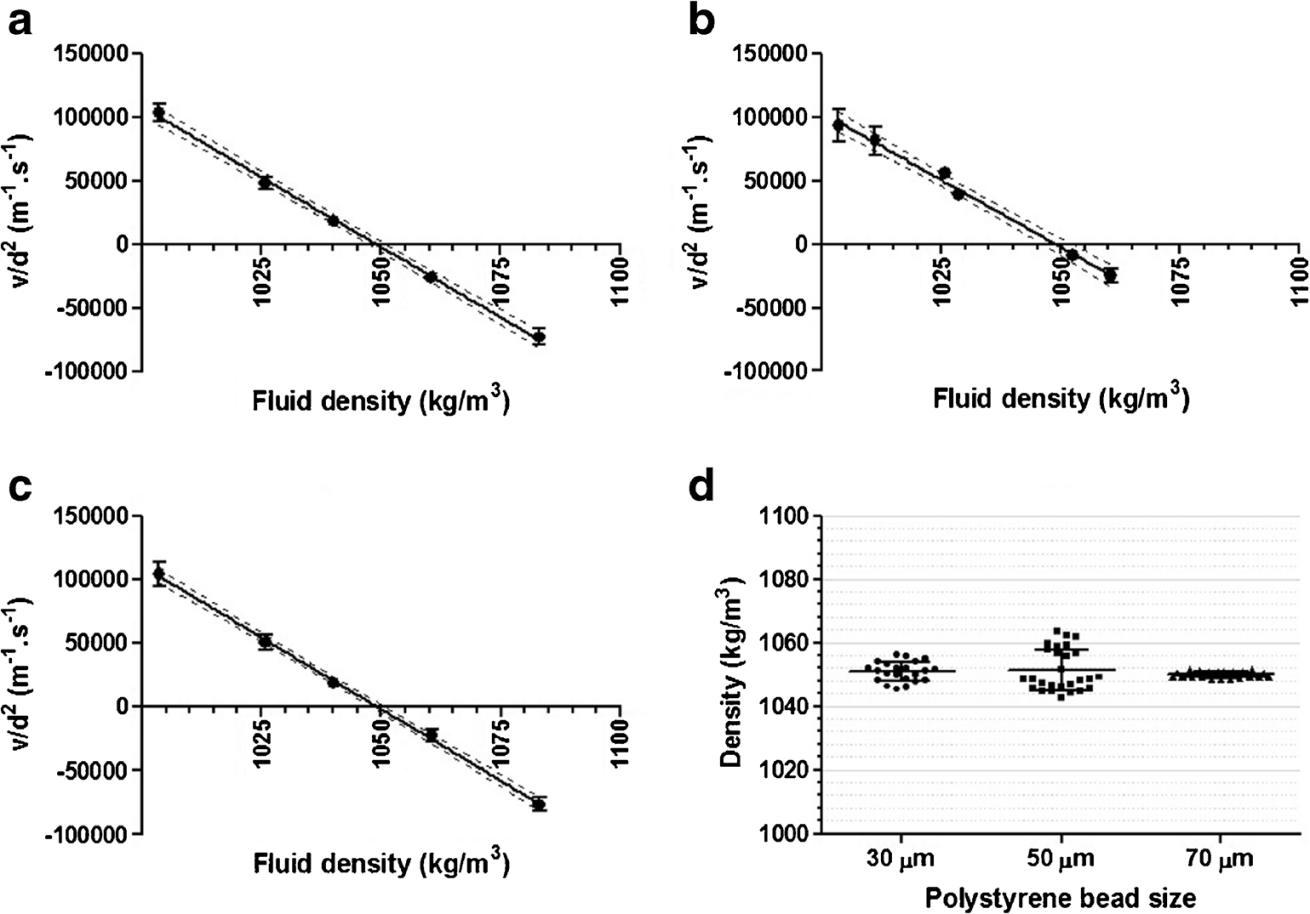
The validation results of the two sedimentation based methods for porosity measurements, using polystyrene size standards. Panels A – C show the relation between fluid density and particle size-normalized sedimentation velocity (v/d^2^) for 30-μm, 50-μm and 70-μm beads, respectively. Each data point represents the average and standard deviation of the v/d^2^ derived from tracking of at least 20 individual particles in the corresponding fluid density. The linear relation between fluid density and sedimentation velocity is denoted as the solid line, with the 95% confidence of interval of the linear relation between the dashed lines. Note that only for 50-μm and 70-μm beads the attained velocities were corrected for the wall effect. Panel D shows density distributions of the investigated polystyrene beads in fluids closest to the nominal polystyrene density (i.e., fluid density of 1040, 1052 and 1060 kg/m^3^, respectively, for 30-, 50- and 70-μm beads).

The same polystyrene beads of different sizes were used to validate the two methods applied in our study. For Method I (the density-matching method), particle sedimentation velocities were determined in liquids with different densities and the results are shown in Fig. 3. The relation between fluid density and particle size-normalized sedimentation velocity (v/d^2^) was shown to be linear (R^2^ above 0.97), with nearly the same slope for all beads (−2206 ± 45 *vs*. -2127 ± 87; 2253 ± 36 for 30-, 50- and 70-μm beads, respectively). Using linear regression on the data points helped finding the intercept with the X-axis, which corresponds to the average density of the corresponding particles. In case of polystyrene beads the derived particle density appeared to be statistically equal to the reference values from the manufacturer (see Table I). Altogether, these results demonstrate the validity of the method.

**Table I:** Densities of Polystyrene Beads Obtained by Sedimentation Velocity Tracking with the Density-Matching Method (Method I) and the Stokes Derived Method (Method II)

Specifications provided by the manufacturer		Sedimentation velocity tracking		
		Method I		Method II
Average size ± SD (μm)	Average density (kg/m^3^)	Average density ± SD (kg/m^3^)^a^	Goodness of fit (R^2^)	Average density ± SD (kg/m^3^)^b^
29.8 ± 0.4	1050	1050 ± 3	0.988	1049 ± 1
50.2 ± 0.5		1049 ± 3	0.968	1049 ± 1
69.1 ± 0.8		1050 ± 2	0.994	1050 ± 1

^a^mean and standard deviation ((SD), derived from measurements in 5–6 different suspending liquids) of the density and the goodness of fit

^b^density distributions (*n* = 20 particles)

For Method II (the Stokes derived method) only the data from a fluid having a density close to the expected bead density was used in order to obtain sedimentation velocity values with the highest possible accuracy. As explained in the “Materials and Methods” section, the individual particle velocities were used to calculate the particle density through Stokes’ law of sedimentation, after corrections for shape and wall effect. The results listed in Table I show that the polystyrene bead densities determined for each bead size were comparable to the specifications from the manufacturer. With Method II the density distribution of the polystyrene beads could be derived, as shown in Fig. 3d. Moreover, from the ratio of the calculated and given density distribution the porosity distributions were derived. The porosity values of polystyrene beads were calculated to be in the range of 0.1 ± 0.1% for 30 and 50 μm beads and 0.0 ± 0.1% for the 70 μm beads, which is in excellent agreement with the expected value of zero for solid beads.

In conclusion, we have established two valid methods to determine microparticle density and porosity based on sedimentation velocity tracking. In addition, with Method II, one is able to derive the density distribution and hence the porosity distribution.

### Sedimentation Velocity Tracking for Determining PLGA Microparticle Porosity

**Fig. 4:**
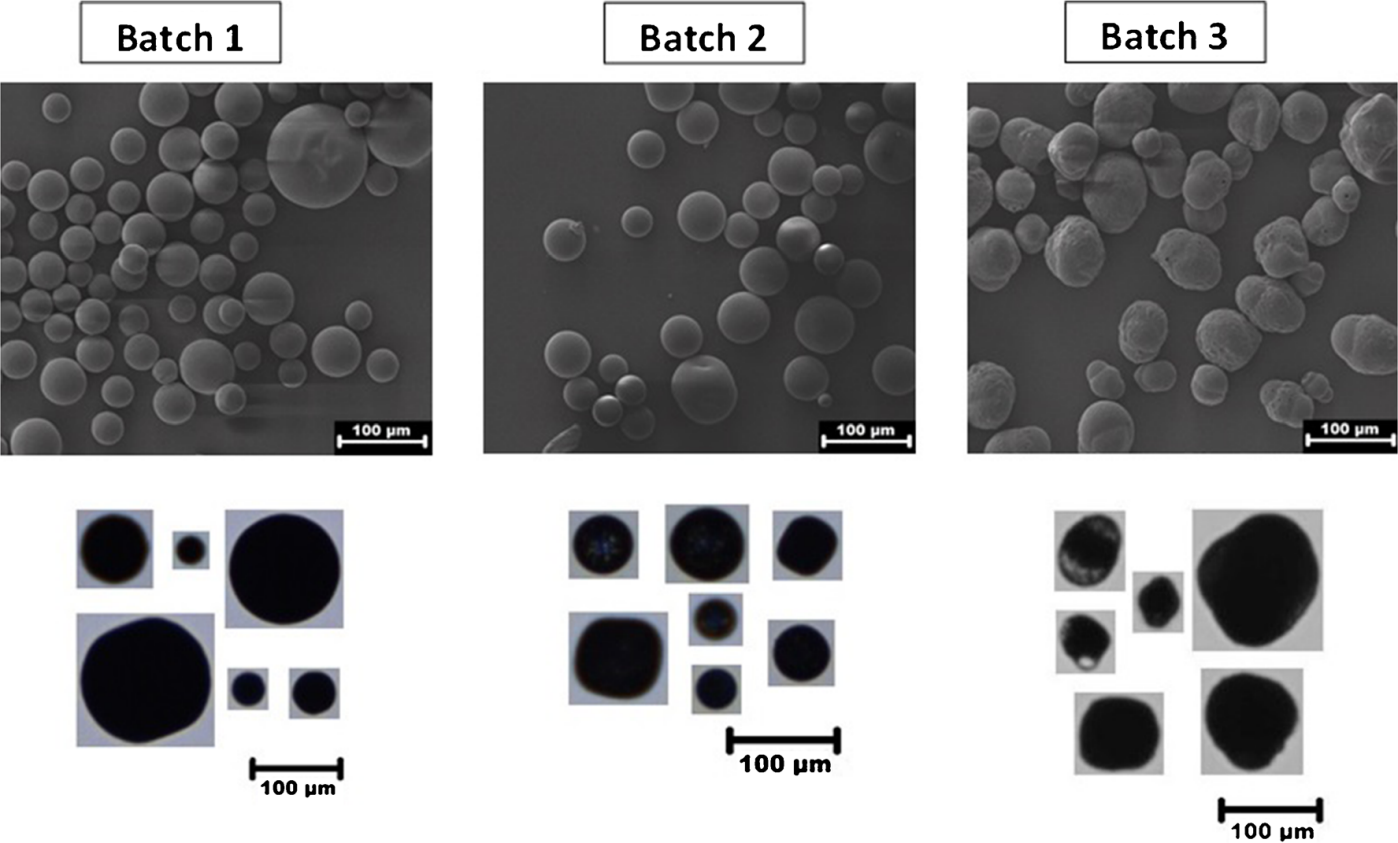
Representative high resolution scanning electron microscopy (upper panels) and FlowCAM images (lower panels) of the different PLGA microparticle batches.

The shape of the PLGA microparticles was studied by using the images and morphological data provided by FlowCAM as well as with SEM imaging (Fig. 4). Batches 1 and 2 appeared to have predominantly spherical particles with a smooth surface, whereas batch 3 contained mainly misshaped particles, with highly irregular surfaces. Also with FlowCAM, these morphological properties were distinguishable, albeit with a less detailed resolution compared to SEM. These differences were apparent from a number of morphological descriptors, in particular aspect ratio, circle fit, circularity, intensity and transparency (Table II).

**Table II:** Morphological Parameters of the Different PLGA Microparticle Batches

Micro-particle batch	Aspect ratio		Circle fit		Circularity		Intensity	
	The ratio of the width/length, where width and length correspond to the minor and major axis of the particle, respectively		Similarity of the particle edge to a best-fit circle, normalized to the range [0,1] where a perfect fit has a value of 1		The circumference of an equivalent area circle divided by the actual perimeter of the particle		The average greyscale of the pixels making up a particle (range = 0–255; 255 is most intense)	
	Mean	SD	Mean	SD	Mean	SD	Mean	SD
1	0.982	0.010	0.959	0.004	0.954	0.006	52.83	3.93
2	0.980	0.007	0.957	0.003	0.927^a^	0.015	45.94	18.26
3	0.862^a^	0.070	0.844^a^	0.067	0.907^a^	0.027	19.80^a^	7.51

^a^Significantly different from the other batch(es) (one-way Anova (*p* < 0.0001) followed by post-hoc Tukey’s multiple comparison test (*p* < 0.05))

**Fig. 5:**
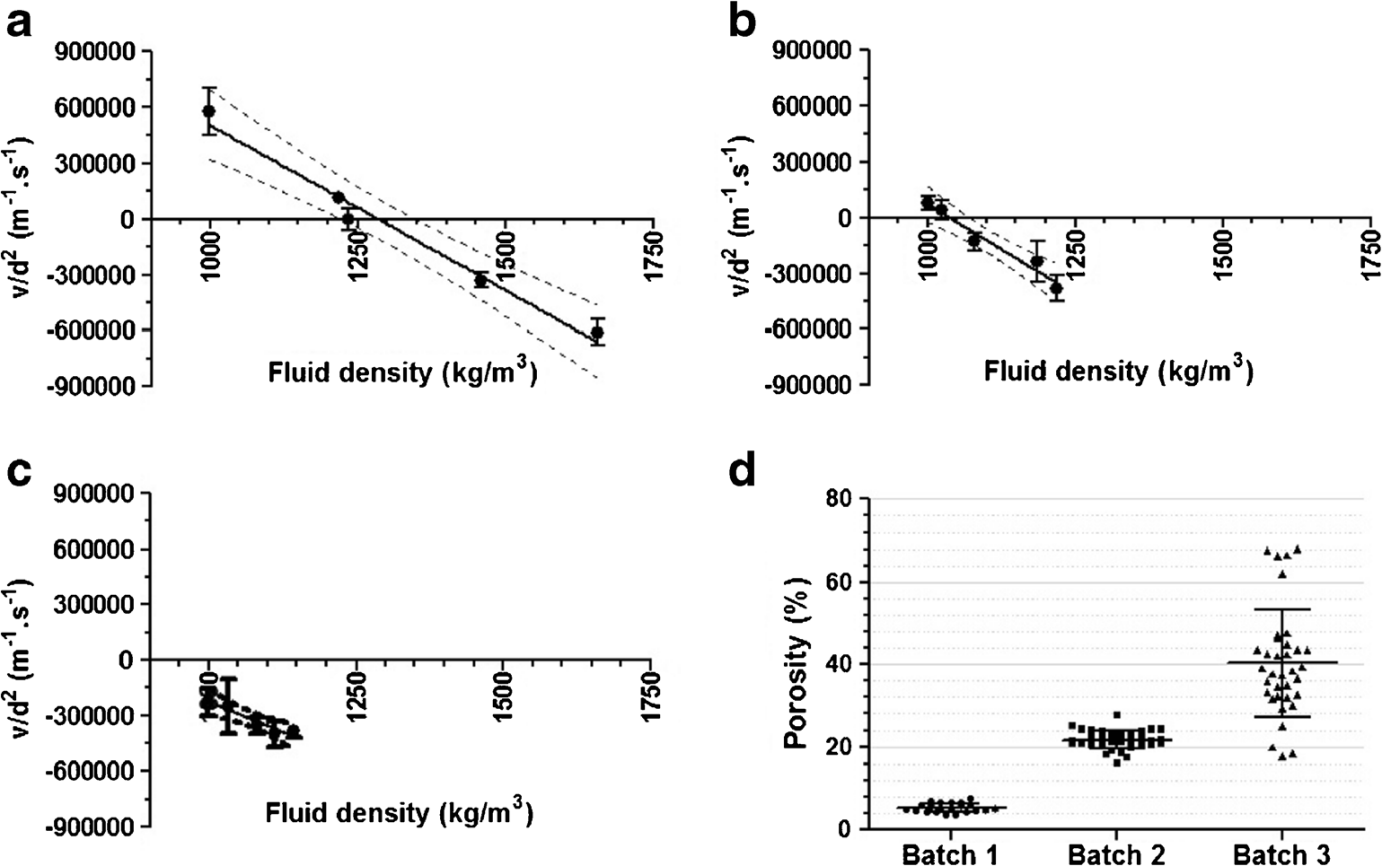
Results of the two sedimentation based methods applied for porosity measurements of the different PLGA microparticle batches. Panels A – C show the relation between fluid density and particle size-normalized sedimentation velocity (v/d^2^) for three different batches (batch 1, 2 and 3, respectively). Each data point represents the average and standard deviation of the sedimentation velocities derived from tracking of at least 20 individual particles in the corresponding fluid density. The linear relation between fluid density and v/d^2^ is denoted as the solid line, with the 95% confidence of interval of the linear relation between the dashed lines. Panel D shows porosity distributions of the investigated PLGA microparticle batches in fluids closest to the nominal PLGA microparticle density (i.e., fluid density of 1233, 1023 and 999 kg/m^3^, respectively, for batch 1, 2 and 3).

The two validated methods were then used to obtain the microparticle porosity, derived from the density (calculated as shown in the “Materials and Methods” section), for 3 different PLGA microparticle batches. As shown in Fig. 5a–c, for each of the batches a linear relation was found between fluid density and average v/d^2^, with a R^2^ value above 0.9. According to the density-matching method (Method I), the regression model for each batch was used to find the X-intercept, representing the average microparticle density, which resulted for all batches in a similar porosity value as measured by MIP (Table III). Noticeably, compared to the other two batches, batch 3 showed a relatively high standard deviation for the average porosity. In addition, the determined porosity of batch 3 tended to be lower than that obtained by MIP.

**Table III:** Densities and Porosities of 3 PLGA Microparticle Batches Obtained By Sedimentation Velocity Tracking with The Density-Matching Method (Method I) and the Stokes Derived Method (Method II)

Micro-particle batch	Mercury intrusion porosimetry		Sedimentation velocity tracking				
			Method I			Method II	
	Porosity (%)	Expected density range (kg/m^3^)^a^	Average density ± SD (kg/m^3^)^b^	Average porosity ± SD (%)^b^	Goodness of fit (R^2^)	Average density ± SD (kg/m^3^)^c^	Average porosity ± SD (%)^c^
1	4.0	1313–1332	1284 ± 43	4.2 ± 3.2	0.979	1270 ± 11	5.2 ± 0.9
2	21.6	1028–1243	1036 ± 31	21.9 ± 2.3	0.960	1039 ± 29	21.6 ± 2.2
3	51.9	626–1135	823 ± 177	39.1 ± 13.3	0.903	801 ± 152	39.8 ± 11.4

^a^calculated from the known solid composition of the PLGA microparticle batch, assuming that the pores were filled with air (lower values) or suspending liquid (upper values)

^b^mean and standard deviation ((SD), derived from measurements in 5–6 different suspending liquids) of the density and the goodness of fit

^c^density or porosity distributions (*n* = 20 particles)

For all batches, the Stokes derived method (Method II) showed similar porosity values as compared to those obtained by Method I. For this purpose the results from the fluid density closest to the matching density value (data point closest to the X-intercept in Figure A-C) were used. Furthermore, for each batch the porosity distribution was derived. As shown in Fig. 5d, a relatively broad porosity distribution was found for batch 3 as compared to the other batches.

## Discussion

With the increasing need for controlled drug release formulations, the characterization of these systems becomes increasingly important. This holds true in particular for the determination of the porosity, for which complex and material-consuming methods such as MIP and gas adsorption are generally used. In our study we have evaluated the feasibility to determine the porosity of individual microparticles from their sedimentation rate in a liquid. Because of its reported applicability for sedimentation rate determination (12), we selected a FlowCAM for our investigation. Tracking of the sedimentation velocity of microparticles becomes possible by obstructing the liquid flow after introduction of the sample into the flow cell of the instrument.

The precision and accuracy of sizing are very important for the purpose of obtaining the correct particle density by sedimentation velocity tracking. Fortunately, the methods developed here do not require the counting (and sizing) of all particles in the suspension, unlike in conventional applications of flow imaging microscopy where particle concentration often is an important output parameter (10). Therefore, we have explored possibilities to use the image-derived data of individual particles to select only particles that are in focus (to allow accurate sizing) and show regular settling behavior (to enable accurate determination of the sedimentation velocity).

This resulted in a number of inclusion criteria that were used as indicators for the aforementioned characteristics (size and settling behavior). The considerable (quadratic) effect of particle diameter on the calculated sedimentation velocity (cf. Eq. 2) was in particular important to consider. In order to decrease potential errors related to this effect, we investigated appropriate instrument settings for our method development. As FlowCAM offers the operator the advantage to choose the magnification, we have tested the influence of magnification magnitude on the sizing precision and accuracy of 5-μm and 20-μm beads in an additional study (Supplementary Figure S3). The outcome of that study indicates that the use of a lower lens magnification improves the sizing precision and accuracy of the instrument. An additional advantage of a lower magnification is that it would result in a larger field of view, thus in a larger sedimentation path that could be tracked, which is beneficial for the accuracy of the sedimentation velocity calculation.

We found the edge gradient parameter to be a useful indicator for focus, and therewith for accuracy of the sizing. The intensity of the outer border of the particle determines the edge gradient value. Here, a low edge gradient number may indicate that the edge of the particle is spread out, as that happens with out-of-focus particles, and a very high value indicates a very sharp contrast at the edge of the particle. All the pixels inside the edge border are eventually used to determine the area of the particles, which in turn is used to calculate the diameter (ABD). Our study indicates that very sharp contrast leads to underestimation of the particle size and therefore both a lower and an upper limit for the edge gradient were included in the particle selection criteria (100 – 200 a.u.). The edge gradient values of the PLGA microparticles were found to lie between 75 and 223. Polystyrene beads showed overall comparable (very dark) greyscale intensity as the PLGA microparticles that we studied. Also the refractive index of PLGA and polystyrene do not differ that much (1.46 and 1.59, respectively). In this case it is valid to consider that the edge gradient range that is chosen based on the polystyrene bead study, would be suitable for the PLGA microparticles as well. In case one wants to study the density (and/or porosity) of more transparent microparticles, the edge gradient range that represents in-focus particles may differ. Furthermore, the focal plane in the flow cell is ideally positioned in the middle of the depth of field of view, where particles in focus are assumed to have the maximum distance from the front and rear wall of the flow cell, hence the lowest resistance effects from these two walls. Despite these measures, one has to realize that the effect of imperfect focus and particle edge definition on the sizing may result in some errors in the density calculations and may be (at least partly) responsible for the distributions found for each group of the presumably uniformly dense polystyrene beads.

The methods we developed here can be deterrent because they may seem to involve time-consuming processes. This holds true for the utilization of the inclusion criteria for a dataset of settling particles and for the procedure in Method I of tracking the sedimentation in different liquids and then finding the apparent density through intrapolation of the linear relation between fluid density and size-normalized sedimentation velocity (v/d^2^). However, the length of this part of the method can be easily reduced to even minutes if a computing toolbox based on a package like MatLab is used to test the named selection criteria on the whole data set and derivation of individual sedimentation rates with subsequent corrections and porosity calculations. This is particularly valuable when the method is used for routine analysis of, e.g., microparticle batches.

Suspending fluids with a higher density than the expected particle density were chosen to confirm the applicability of the method for particles with a relatively low density, such as highly porous PLGA microparticles. Such particles would move upwards (float) in conventional suspending media, resulting in negative sedimentation velocity values. In the experiments where polystyrene beads of different sizes were found to be floating, the calculated densities were the same as provided by the manufacturer. This highlights the applicability of this method for a wide range of particle sizes, densities and porosities. The altered conditions in our study concerned fluid density, but theoretically fluid viscosity could be another parameter to adjust.

For the density calculations using Stokes’ formula we have only used the data of particles in fluids with a density close to the particle density. In addition, as seen from Fig. 1, the precision of the sedimentation velocity determination increases when the particle density is close to the fluid density. A significant added value of Method II over Method I lies in the fact that the final mean and standard deviation of the density (and hence porosity) from second approach resembles the population mean and the variability in it. In Method I the averages and standard deviations of different populations in different liquids are used to calculate a mean and standard deviation of the batch density (and porosity).

One has to realize that the first raw outcome of the approaches presented in this study is the sedimentation velocity of particles, which is proportional to the apparent density of the particle. Therefore, the method is a reliable approach for measurement of the density of the particles as evidenced by investigation of the standard polystyrene beads. Deduction of porosity from the apparent density needs some assumptions, the most important of which concerns the filling of the pores, with air or suspending fluid.

In a preliminary study we have investigated the effect of the time lag (up to 4 days) between the preparation of the microparticle suspension (batch 3) and the measurement. We found that the equilibration time did influence the density of the particles, especially after 24 h of incubation. This may be caused by a change of pore properties due to release of the API and potential degradation of the PLGA. Therefore, we fixed the equilibration time to 3 h, which should be long enough for wetting of the surface of the particles and short enough to avoid major changes in the pore properties.

Considering the hydrophobic properties of the PLGA and the use of aqueous suspending media, one can expect that the diffusion of water through a primarily hydrophobic matrix would be considerably delayed. The assumption of air-filled pores for the porosity calculations appeared to be fair when the MIP data was compared to the porosity calculations based on flow imaging experiments. The results summarized in Table III indicate that the obtained densities of the PLGA particles from all batches are closer to those calculated from the MIP porosity data when pores are assumed to be filled with air. It has to be realized, however, that partial filling of the pores with liquid cannot be totally ruled out and it may be a source of the relatively large SD for batch 3 compared to the other 2 batches.

Due to low nominal density of some PLGA particle batches and the presence of air-filled pores, getting a particle density higher than the density of water was not possible for all particles and therefore conditions for particle settling could not always be achieved, not even in absence of cesium chloride. Decreasing density of an aqueous solution can be realized by addition of alcohols. However, it was observed in a small experimental trial that the presence of ethanol caused aggregation of microparticles. Nevertheless, velocity measurements in different fluids showed a good linear relation, in terms of R^2^ values, in particular for the first two batches, suggesting that an extrapolation of the data points to obtain the intercept would be justified. PLGA microparticle batch 3 showed the lowest degree of linear fit and the lowest precision of the density and porosity determination. This may be due to the highly uneven shape and surface of these microparticles compared to the others, as well as the much higher porosity. Nevertheless, the obtained porosity was similar to the value obtained by MIP and significantly different (*p* < 0.05) from the porosities obtained for the other tested PLGA microparticles.

With Method II sedimentation of individual particles is measured (therewith porosity of individual particles) and the mean and standard deviation of the investigated particle population is calculated. The larger relative standard deviation in the density (and hence porosity) that is seen for batch 3 may also be considered as an existing wide porosity distribution for this specific batch. Therefore, the second approach provides better insight into the porosity details on particle level, which is an advantage over Method I and the conventional methods for porosity determinations.

## Conclusion

In conclusion, we have developed, optimized and validated two sedimentation velocity tracking methods to assess the porosity of micron sized particles. For this purpose we used a FlowCAM instrument, but it is expected that the methods can be transferred to other flow imaging microscopes as well. For three batches of PLGA microparticles widely differing in porosity, both methods yielded porosity values that were similar to the values obtained by MIP, while requiring up to 100-fold smaller amounts of material. The methods could therefore be useful as a viable alternative to conventional methods for determining microparticle density and porosity.

## Electronic supplementary material

Below is the link to the electronic supplementary material.

ESM 1 (DOCX 462 kb)

## Abbreviations

ABD: Area based diameter
API: Active pharmaceutical ingredient
FDA: Food and Drug Administration
FOV: Field of view
MIP: Mercury intrusion porosimetry
PBS: Phosphate buffered saline
PBS-T: PBS containing 0.01% (*w*/*v*) polysorbate 80
PLGA: Poly lactic-co-glycolic acid
SEM: Scanning electron microscopy
