# Supplementary material for: Determination of the Porosity of PLGA Microparticles by Tracking Their Sedimentation Velocity Using a Flow Imaging Microscope (FlowCAM)
Source: Pharm Res. 2017 Feb 17;34(5):1104–14. doi: 10.1007/s11095-017-2120-8 (PMC5382175; doi:10.1007/s11095-017-2120-8)
Supplement: Supplementary file 1 — (DOCX 462 kb) [file 11095_2017_2120_MOESM1_ESM.docx]

**Supplementary Figures**

**Determination of the porosity of PLGA microparticles by tracking their sedimentation velocity using a flow imaging microscope (FlowCAM)**

A.S. Sediq^1^, S.K.D. Waasdorp^1^,M.R. Nejadnik^1^, M.M.C. van Beers^1,2^, J. Meulenaar^2^, R. Verrijk^2^, W. Jiskoot^1,^*

^1^ Division of Drug Delivery Technology, Cluster BioTherapeutics, Leiden Academic Centre for Drug Research (LACDR), Leiden University, Leiden, the Netherlands

^2^ Dr. Reddy’s Laboratories Ltd., IPDO Leiden, the Netherlands.

* Corresponding author:

Wim Jiskoot

Email: w.jiskoot@lacdr.leidenuniv.nl

Phone: +31 71 527 4314

Fax: +31 71 527 4565

**
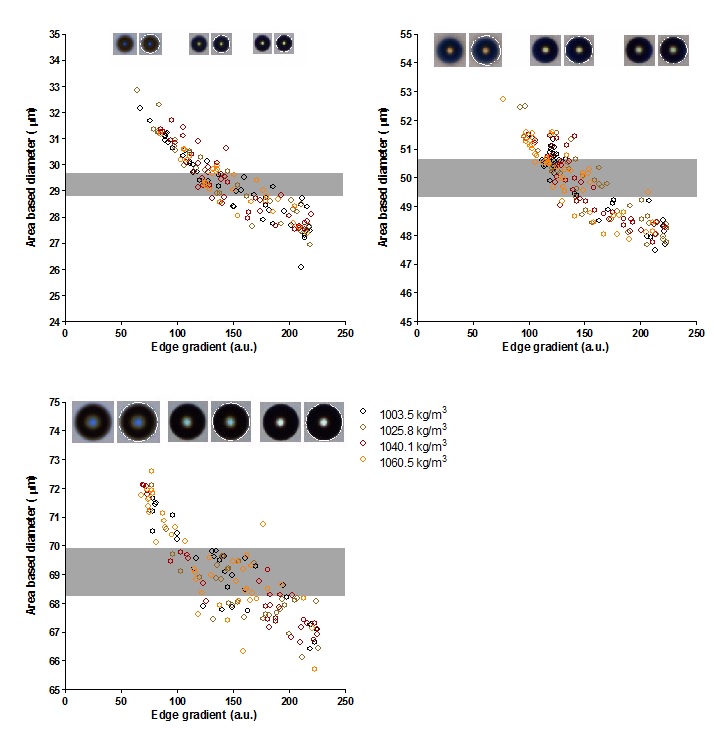
Supplementary Figure S1:** Area based diameter (ABD) of the three tested polystyrene beads plotted against the corresponding edge gradient value. The color of each open circle represents a single particle analyzed in a suspending medium having the density as indicated in the legend. The grey bar indicates the average and standard deviation of the size as stated by the manufacturer. Images on top of the graphs are deduced from the analysis, each pair corresponding to a particle with the lowest (left pairs) medium (middle pairs) and highest (right pairs) edge gradient values. The paired images show on the left the original image of a particle and on the right an image with an edge trace. The edge gradient value provided by VisualSpreadsheet is the average intensity of the pixels making up the outside border of a particle. A low edge gradient number indicates that the edge of the particle is spread out, as that happens with out-of-focus particles, and a very high value indicates a very sharp contrast at the edge of the particle. The results from Figure 1 indicate that the edge gradient value between 100 and 200 corresponds with an ABD value within the range of specifications. Therefore, the edge gradient value was included as one of the criteria for selection of particles for the calculations applied further. Furthermore, the focal plane in the flow cell is ideally positioned in the middle of the depth of field of view, where particles in focus are assumed to have the maximum distance from the front and rear wall of the flow cell, hence the lowest resistance effects from these two walls.

**
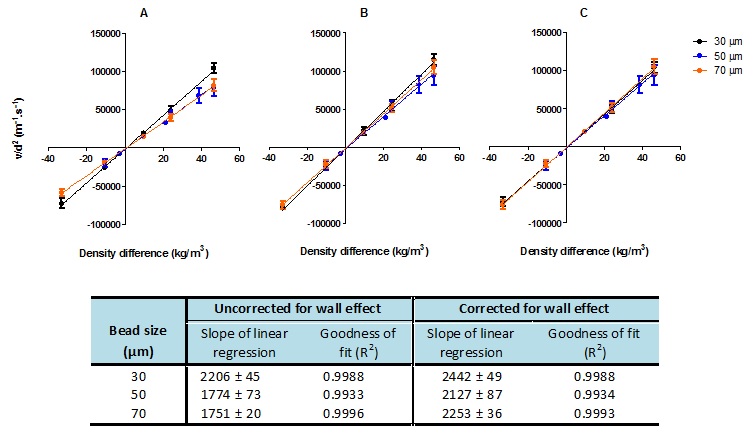
**

**Supplementary Figure S2**: Average and standard deviation of the measured v/d^2^ for different beads in liquids with different densities: 30-µm beads (black circles) 50 µm-beads (blue circles) and 70-µm beads (orange circles). Wall effect correction was (A) not applied, (B) applied for all beads, and (C) applied for 50- and 70-µm beads. In each graph the data points were fitted by linear regression. The slopes and R^2^ values of each linear regression are shown in the table. It is seen that the slopes of the linear regression depends on the bead size, despite the use of v/d^2^. When wall correction is applied for the two largest bead sizes, the slopes become similar. Therefore, based on this study we chose to apply wall correction only for the microparticles with the size equal to or higher than 50 µm.

**
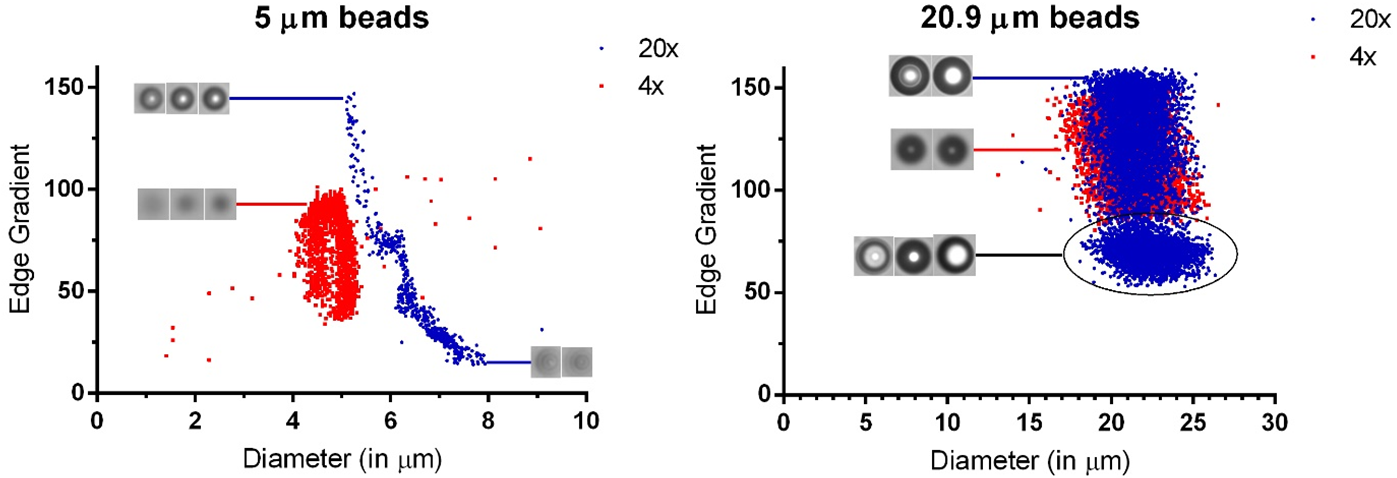
**

**Supplementary Figure S3:** Scatter plot showing the relation between edge gradient and area based diameter of 5-µm (left) and 20-µm (right) polystyrene beads (Thermo Scientific, Fremont, CA, USA), with different magnification lenses (i.e., 20x and 4x). The inserts of particle images show a number of representative particles in appointed parts of the scatter plot.
